# Non-Genetic Healthcare Providers’ Experiences and Perspectives with Rapid Genome-Wide Sequencing in Canadian Neonatal Intensive Care Units

**DOI:** 10.3390/children11080910

**Published:** 2024-07-28

**Authors:** Lauren Piers, Tasha Wainstein, Gustavo Pelligra, Horacio Osiovich, GenCOUNSEL Study, Alison M. Elliott

**Affiliations:** 1 Department of Medical Genetics, Faculty of Medicine, University of British Columbia, Vancouver, BC V6H 3N1, Canada; 2 BC Children’s Hospital Research Institute, Vancouver, BC V5Z 4H4, Canada; 3 Department of Pediatrics, Victoria General Hospital, Island Health Authority, Victoria, BC V8Z 6R5, Canada; 4 Department of Pediatrics, Faculty of Medicine, University of British Columbia, Vancouver, BC V6H 0B3, Canada; 5 Women’s Health Research Institute, Vancouver, BC V6H 3N1, Canada

**Keywords:** rapid genome-wide sequencing, neonatal intensive care unit (NICU), multidisciplinary care, genetic counselling, implementation

## Abstract

Background/Objectives: Rapid genome-wide sequencing (rGWS) continues to transform the care provided to infants with genetic conditions in neonatal intensive care units (NICUs). Previous research has demonstrated that rGWS has immense benefits on patient care; however, little is known about non-genetic healthcare providers’ (HCPs) experiences and perspectives of working with rGWS and supporting families through the rGWS testing process in Canadian NICU facilities. To address this gap, we surveyed and conducted semi-structured interviews with non-genetic HCPs of diverse professions from NICUs in British Columbia. Methods: An interpretive description approach was used to analyze interview transcripts to identify patterns and variations in non-genetic HCPs’ experiences and perceptions with rGWS. Results: Participants had varying degrees of exposure to rGWS and levels of comfort with the testing process. Numerous barriers affecting the implementation of rGWS were identified, including low levels of comprehension of rGWS, longer turn-around times than expected, and having to apply for provincial government approval to access testing. Participants desired more education on rGWS, clear guidelines on the use of rGWS in NICUs, and resources for non-genetic HCPs and parents to support implementation. Conclusions: The results from this study can inform the development of workflows and educational resources on the use of rGWS in NICUs, helping to ensure that the NICU team is supported to optimize rGWS implementation.

## 1. Introduction

Genetic diseases are a significant cause of infant morbidity, mortality, and admission to neonatal intensive care units (NICUs) in Canada [1]. According to Statistics Canada, congenital malformations, deformations, and chromosomal abnormalities have been the leading causes of infant death since 2000, many of which can be attributed to genetic etiologies [2]. Traditionally, genetic testing approaches for critically ill neonates involve candidate gene or serial multi-gene panel testing [3,4]. These approaches are time-consuming; genetic diseases are challenging to identify in NICU patients due to genetic heterogeneity and variable expressivity, thereby prolonging their diagnostic odyssey [3,4,5]. Rapid genome-wide sequencing (rGWS), which includes whole exome and whole genome sequencing, can detect disease-causing genomic variants with a quick turn-around time typically ranging from 3 to 23 days [4,6,7,8,9,10,11,12]. Further, this technology has a diagnostic rate between 30 and 60% when employed in NICU patients suspected of genetic disease [4,7,8,9,10,11,12,13]. Typically, rGWS in NICUs is trio-based, where both biological parents also submit a sample along with the blood sample from the critically ill neonate to aid in the interpretation of genomic variants. A diagnosis from rGWS can alter the medical management of NICU patients, improving health outcomes, preventing morbidity, or expediting the involvement of palliative care for neonates with a life-limiting diagnosis [4,7,8,9,10,11,12,13]. Some challenges remain with respect to the use of rGWS in this setting, such as those related to the interpretation of variants of uncertain significance, and ethical concerns regarding secondary findings.

Previous research has also demonstrated that rGWS has positive impacts on parental experiences in NICUs [13,14,15]. Parents have expressed that receiving rGWS results for their infant reduced their feelings of uncertainty, provided them with a sense of relief, and alleviated their guilt by increasing their knowledge of their infant’s condition [14]. Parents whose infant received a diagnosis through rGWS have indicated that the results helped them plan for their child’s future [13,15] and cultivate connections with families undergoing similar experiences [13]. However, parents considering rGWS for their neonate also experience heightened anxiety, overwhelm, and decisional conflict [16], and higher rates of depression compared to the general population [17]. Further, receiving a diagnosis from rGWS in a pediatric setting has been associated with reduced family functioning and relationships [18]. As the clinical benefits of rGWS support the standard use of this technology in NICUs, these findings highlight the importance of effectively integrating rGWS into NICUs in a way that supports families through stressful life experiences.

In NICUs, healthcare providers (HCPs) work collaboratively as a multidisciplinary team to care for critically ill neonates and their families [19]. The effective integration of rGWS into NICUs requires that the perspectives of all members of the multidisciplinary team are considered to gain a holistic understanding of the barriers and facilitators associated with the clinical implementation of rGWS. Franck et al. (2021) previously conducted a study exploring the perspectives of members of a multidisciplinary team on the use of rGWS to care for critically ill children in five Pediatric Intensive Care Units (PICUs) in California [20]. While all members of the multidisciplinary team supported the use of rGWS in clinical care, there was concern about the team’s lack of knowledge of genomic medicine [20]. For example, many clinicians did not feel competent in their understanding of rGWS test results or in how to communicate the results with families [20]. Although this study highlighted critical barriers experienced by HCPs using rGWS in PICUs, the perspectives of many non-genetic HCPs who work in NICUs, including nurse practitioners, nurses, dieticians, and respiratory, physical, and occupational therapists were not explored. All of these HCPs have unique and critical roles in caring for neonates and their families in NICUs [19]. Therefore, it is essential to understand the experiences of all NICU team members so that their support and education can be optimized to deliver an efficient, evidence-informed clinical service.

In this study, we evaluated what barriers and challenges non-genetic HCPs have encountered when accessing and implementing rGWS in the NICU and what impacts this has had on their support of NICU families. Understanding these barriers can help to inform the development of workflows and educational resources for rGWS in Canadian NICUs, providing support and education to non-genetic HCPs implementing or caring for neonates who undergo rGWS and their families.

## 2. Materials and Methods

A constructivist paradigm was used to inform the design of this qualitative study. This paradigm acknowledges that individual experiences are subjective and may vary between research participants [21,22]. It utilizes inductive methodology to help understand and make meaning of individuals’ experiences in the context of the research question. [21,22]. We therefore did not make use of a pre-existing model or theory upon which to frame our analysis. The interpretation of the participants’ individual experiences was influenced by the subjective experiences and positionalities of the research team. While this research was conducted, LP was a genetic counseling graduate student. Both TW and AE are genetic counselors while HO and GP are neonatologists. All members of the research team have a range of both clinical and research experience, from 2 to >20 years. Further, all members of the research team reside and practice within the province of BC, Canada.

### 2.1. Participants and Recruitment

This study was undertaken at the NICUs at BC Women’s Hospital (BCWH) in Vancouver, and Victoria General Hospital (VGH) in Victoria. Both NICUs are tertiary care centers located in BC, Canada and collectively provide care to approximately 2200 infants per year. Neonatologists, neonatal fellows, pediatric residents, bedside nurses, neonatal nurse practitioners, physiotherapists, occupational therapists, respiratory therapists, dieticians, and social workers were invited to participate in the study. To ensure a broad representation of HCPs, a combination of purposive, convenience, and snowball sampling was used to recruit participants.

### 2.2. Data Collection

Participation in this study consisted of two parts: completion of an online demographic survey followed by a one-on-one semi-structured interview. The demographic survey was completed through the REDCap database [23], housed at the BC Children’s Hospital Research Institute. Participants were asked to indicate the following demographic information: profession, education level, number of years since completing their training program, self-reported genomic literacy, formal training received in genomics, and how often they have provided care to neonates who have undergone rGWS (Supplemental Materials S1). Only those who specified at the end of the survey that they wanted to participate in an interview were contacted for an interview.

Semi-structured interviews were carried out with participants who responded to the interview invitation and consented to participate. Interviews were conducted by LP via video conference (Zoom) and audio-recorded, between December 2022 and February 2024. An interview guide was created by the study team based on the clinical expertise of the researchers (genetic counsellors and neonatologists) and the previous literature. The interview guide (Supplemental Materials S2) was adapted for the different HCPs so that participants were asked questions that aligned with their role and responsibilities in the NICU. The interviews explored participants’ comfort with rGWS, barriers and facilitators they experience when using rGWS in NICUs or supporting families through the rGWS testing process, as well as their perspectives on the clinical and ethical aspects of rGWS in NICUs. Interviews were transcribed verbatim either by a member of the research team or a professional transcription service, Transcript Heroes.

### 2.3. Data Analysis

An interpretive description approach was used to analyze the interview transcripts. Interpretive description allows for deep exploration of research questions and facilitates, generating salient recommendations for practice and policy in a clinical setting in the context of a constructivist paradigm [24,25,26,27]. This methodology was designed for health sciences disciplines and allows for the identification of diverse themes and variations within those themes in relation to a health research question [24,25,26,27]. The transcripts were coded by LP using phronetic iterative analysis to guide the coding process [28,29]. The transcripts were coded line by line into basic descriptive units of content using an inductive approach, which involves generating codes based on the data rather than using a pre-established derived set of codes. Once the first five transcripts were coded, LP generated a primary codebook to facilitate collaborative analysis among the research team [30]. The research team met regularly to construct an understanding of the data and review the primary codebook. The primary codebook was applied iteratively to subsequent transcripts as they became available for analysis and was updated as additional transcripts were coded. The transcripts were constantly compared to one another to ensure consistency in the application of the primary codes within and between transcripts [31].

Upon the completion of primary coding, axial coding was employed to group together similar codes into umbrella categories and conceptually connect codes together to create network maps [28,29]. Field notes and memos that were written after each interview and team meeting were used to help inform this stage of data analysis. The research team met to collaboratively interpret the main concepts in the data as well as the factors/explanations that contributed to each concept and the connections between them [22,28,29]. This allowed for the identification of common ideas in non-genetic HCPs’ experiences with rGWS in the NICU, as well as any unique and contrasting ideas amongst the participants. Participants were recruited until the research team assessed the sample size to have sufficient informational power [32] to address the research question. Based on the narrow aim of the study; the diversity of the sample with respect to profession, years of experience, and education level; and the high quality of data collected during interviews, data collection was considered to be completed after 11 interviews were conducted [32]. Our research also demonstrates theoretical sufficiency [33], in that we have constructed an analysis that is adequate in terms of addressing the research question.

## 3. Results

### 3.1. Demographics

The demographic survey was distributed to 118 individuals who met the eligibility criteria. A total of 30 non-genetic HCPs from BCWH and VGH completed the survey: 19 bedside nurses, three neonatologists, two medical residents/fellows, two respiratory therapists (RTs), one occupational therapist (OT), one physical therapist (PT), and two social workers. Table 1 presents the complete demographic information from survey respondents. The survey response rate was 25.4%.

Out of the 30 survey responses, 16 participants indicated a willingness to be interviewed. A total of 11 interviews were conducted (six nurses, three neonatologists, one RT, and one social worker; eight from BCCH and three from VGH). Interviews ranged in length from 24 to 43 min (average 30 min).

**Figure 1:**
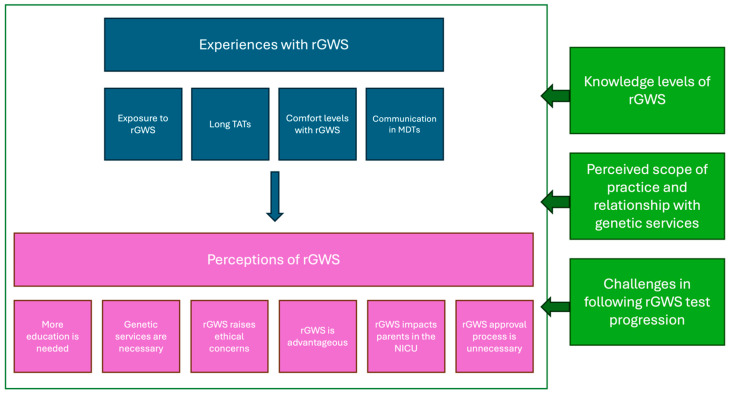
Organizational depiction of the non-genetic HCPs’ experiences with rGWS (blue), perceptions of rGWS (pink), and the factors that influenced their experiences and perspectives (green).

Our analysis reveals the broad range of participants’ experiences with and perceptions of rGWS. We also demonstrate the factors that give rise to these experiences and perceptions (Figure 1).

Representative quotations for all aspects of the analysis appear in Table 2. Quotations are labeled in text using an alphanumeric code (e.g., E1) which indicates their location within Table 2.

### 3.2. Experiences with rGWS

#### 3.2.1. Exposure to rGWS/Genetics

Participants had varying degrees of prior exposure to genetics and rGWS. The majority of participants did not have formal training in genetics (Table 1). Additionally, participants had different levels of exposure to rGWS in the NICU, ranging from no prior exposure to frequent exposure (Table 1). Some neonatologists noted an increased uptake in rGWS in the NICU over the past few years, leading to more frequent exposure to infants undergoing rGWS. The social worker reported being frequently involved in providing support to families once results from rGWS had been received. Others, primarily nurses, reported that they experienced infrequent involvement in caring for neonates with genetic conditions or infants who underwent rGWS (E1). This resulted in barriers to caring for patients with genetic conditions due to unfamiliarity with rGWS.

#### 3.2.2. Long Turn-Around Times

Although the reduced time frame to receive rGWS results was noted by the participants as an advantage, the majority expressed having to wait longer than expected to receive results. Most participants described that the turn-around time (TAT) exceeded the expected seven days. In some instances, participants noted that it took weeks to receive the results. Delays in TATs were burdensome as the participants had to continually follow up with the genetics team (throughout the text, “team” represents genetic counsellors and/or clinical geneticists) about when the results were expected (E2).

#### 3.2.3. Level of Comfort with rGWS

Participants described various levels of comfort with the rGWS testing process, which included discerning which infants should undergo rGWS, obtaining consent, interpreting results, disclosing results to families, and providing emotional support to families. A general trend emerged among the neonatologists that they felt comfortable with specific components of the rGWS testing process under straightforward circumstances (E3) but felt less comfortable disclosing complex results, including incidental findings, secondary findings, or variants of uncertain significance (VUSs) (E4). Comfort levels for other elements of the rGWS process were not situation-dependent. Some neonatologists expressed general discomfort in ordering rGWS but felt comfortable obtaining consent from families.

Overall, the nurses and the RT described feeling uncomfortable answering families’ questions about rGWS (E5). As a result, they had to direct the parents’ questions toward the genetics team or follow up with the genetics team themselves to find answers to their questions. Further, the majority of the nurses articulated that collecting the blood sample required for rGWS was a challenging process because the logistical details and sample requirements were not clearly laid out. The majority of nurses expressed feeling comfortable being involved in modifying patient care based on rGWS results.

The social worker described feeling comfortable providing emotional support to families undergoing rGWS. However, they expressed that it was more difficult to help families navigate negative results compared to positive results as non-diagnostic results can cause uncertainty, making it challenging for families to plan for the future.

#### 3.2.4. Communication Surrounding rGWS with the Multidisciplinary Team

Many participants described experiences of being involved in conversations about rGWS with their multidisciplinary team (MDT). Some of these participants described instances of effective communication about neonates who had undergone rGWS, enhancing patient care. In particular, all of the neonatologists and some nurses described the genetics services at their hospital as accessible, providing them with support when caring for infants undergoing rGWS. Some nurses, the RT, and the social worker described being explicitly informed of their patient’s rGWS results by genetics or neonatology during patient rounds or MDT meetings, which allowed them to gain a greater understanding of how to care for their patient or provide families with emotional support (E6). However, some nurses and the RT also described experiences of poor communication about rGWS with their MDT (E7).

### 3.3. Perceptions of rGWS

#### 3.3.1. More Education Is Needed

All participants expressed a need for more education about rGWS and genetics to increase their knowledge and comfort. Participants across all specialties articulated that more knowledge would provide them with insights about how to better care for their patients and families (P1). Some participants expressed that more education would increase their capacity to support parents by answering their questions about rGWS and allow them to be more empathetic towards families undergoing rGWS. Further, multiple participants described being fascinated with genetics or genetic technology, indicating a genuine desire to learn more (P2).

#### 3.3.2. Genetic Services Are Necessary

Participants across various disciplines discussed the importance of involving geneticists and genetic counselors in caring for patients requiring rGWS (P3). The neonatologists highlighted that rGWS is ordered through consultation with the medical genetics team. Non-genetic HCPs relied on the genetics team for the implementation of rGWS as they believed they had additional expertise in discerning the most appropriate genetic test, counseling families about genetic test results, and providing information on the best management for rare genetic syndromes (P4).

#### 3.3.3. rGWS Raises Ethical Concerns

There were various perceived ethical concerns surrounding the use of rGWS in NICUs. Many concerns centered around secondary and incidental findings for adult-onset conditions (P5). Others were concerned that VUSs may induce parental anxiety (P6).

#### 3.3.4. rGWS Is Advantageous

Despite the perceived ethical concerns, participants perceived that rGWS has many advantages. The main perceived advantage was a faster TAT compared to conventional testing, allowing for timely changes in management and reducing time in the NICU (P7). A fast TAT was perceived as a significant advantage by the social worker as receiving results quickly allowed them to provide families with emotional support while their neonate was still admitted to the NICU (P8).

#### 3.3.5. rGWS Impacts Parents in the NICU

Participants believed that rGWS had positive and negative psychological implications on parental well-being. Multiple participants were concerned that secondary findings and VUSs induced parental anxiety. Participants were cognizant that NICU parents already experience intense anxiety from being in a stressful environment, making them concerned that rGWS introduces additional sources of worry for vulnerable parents (P9). Conversely, many participants noted positive impacts of rGWS on parental mental health, such as the ability to provide parents with answers, clarity about the future, and relief (P10).

#### 3.3.6. Approval Process Is Unnecessary

Some participants commented on the requirement of having to apply for government funding and approval for rGWS prior to ordering the test. These participants questioned the necessity of having to apply for funding as all critically ill infants are ultimately approved for testing (P11). Others expressed frustration that having to apply for funding delays the initiation of time-sensitive testing, defeating the purpose of having a rapid test with a reduced TAT (P12).

### 3.4. Factors Influencing HCPs Experiences and Perspectives with rGWS

Three factors were found to influence the participants’ experiences and perspectives with rGWS: (1) their knowledge level of rGWS, (2) their perceived scope of practice and relationship with genetic services, and (3) the challenges in following rGWS test progression.

#### 3.4.1. Knowledge Level about rGWS

All participants expressed gaps in their knowledge about rGWS. Overall, the neonatologists possessed greater knowledge about rGWS, the scope of the test, and the types of results generated by rGWS. However, there were still components of rGWS with which the neonatologists were unfamiliar (F1).

The majority of the nurses and the RT articulated that their overall comprehension level of rGWS was low (F2). Many of these participants had a limited understanding of the scope of the test, the logistics for collecting the blood sample, and the potential types of results (F3).

#### 3.4.2. Perceived Scope of Practice and Relationship with Genetic Services

Many participants believed that implementing rGWS and supporting families through testing fell primarily within the scope of practice of medical genetics teams rather than within their own scope. Multiple participants expressed that genetics teams should lead the implementation of rGWS in NICUs because of their deeper understanding of rGWS. Additionally, many participants developed a collaborative relationship with the genetics team at their hospital. This led to participants being able to readily access support from their genetics team when they were unsure about how to care for neonates with suspected genetic conditions or had questions about rGWS. Because of this, many participants believed that it did not make sense to provide care to infants undergoing rGWS without involvement from their genetics team.

One neonatologist expressed that since the genetics team is responsible for ordering rGWS, they should be involved in all aspects of patient care related to rGWS until the case is finished. Another neonatologist believed that they should be the second professional to support families through the rGWS process after the genetics team (F4).

The nurses perceived blood sample collection for rGWS to be within their scope of practice (F5). However, aside from sample collection, the nurses expressed that many elements of rGWS were outside of their scope, including obtaining consent from families for rGWS, disclosing or explaining results, applying for funding, and directing management based on rGWS results (F6).

Some participants (like nurses and the social worker) believed that once rGWS results were received, it fell within their scope of practice to educate themselves on the results to support the family regarding planning for the future, the impact of test results on NICU stay duration, mental well-being, and accessing additional supportive resources (F7). Some participants described having to wait an extended amount of time for rGWS results, requiring them to follow up with genetics to inquire if funding was approved (F8).

### 3.5. Strategies for Effective rGWS Implementation

**Figure 2:**
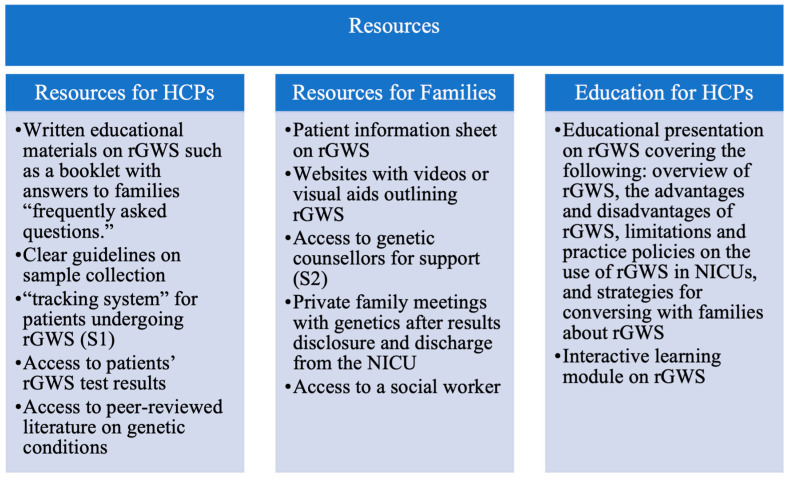
Suggested HCP and family-centered resources and educational strategies to help promote effective implementation of rGWS in NICUs.

Participants agreed that developing guidance and resources to inform the use of rGWS in NICUs would be helpful. Three main strategies were shared by participants to help effectively implement rGWS into NICUs and ensure that families are supported: (1) developing resources for HCPs (S1), (2) providing families with resources (S2), and (3) educating non-genetic HCPs about rGWS (S3). Participants’ specific suggestions for HCP resources, family resources, and HCP education are displayed in Figure 2. Table 2 presents the critical elements that should be addressed for the effective implementation of rGWS in the NICU as suggested by the participants.

In addition, we make recommendations that could help inform a guideline with respect to the integration of rGWS in Canadian NICUs (Table 3).

## 4. Discussion

This study provides unique insight into the experiences and perspectives of rGWS among non-genetic HCPs with diverse roles in NICUs. To our knowledge, this is the first study to offer an in-depth analysis of the experiences and perspectives of nurses, social workers, and respiratory therapists with rGWS in NICUs. The perspectives of nurses are especially important to understand as they are the frontline and primary caregivers in NICUs [19].

The results from this study highlight that a lack of knowledge about rGWS is a key barrier experienced by non-genetic HCPs when implementing rGWS and supporting families through rGWS in NICUs. HCPs’ knowledge levels about genomic medicine and sequencing have previously been described as a central factor influencing the successful integration of rGWS into NICUs [20]. In the current study, a limited understanding of rGWS fueled discomfort with many elements of the rGWS testing process among participants. Some participants became less comfortable with rGWS as the complexity level of patient cases and rGWS results increased. This finding is similar to what was reported by Franck et al. (2021), who found that HCPs in the NICU felt more comfortable communicating negative rGWS results to parents but were more likely to consult genetics experts when positive or incidental findings on rGWS results were reported [20].

All participants identified education on rGWS and the development of resources for non-genetic HCPs as critical strategies to address knowledge gaps and feelings of discomfort, and promote the effective implementation of rGWS in NICUs. The need to improve the genomic literacy of non-genetic HCPs to help address integration barriers of genomic sequencing into clinical practice is well described in previous studies [20,34,35,36,37]. Bupp et al. (2023) developed a successful model for rGWS implementation in NICUs, which included a genomics course on testing protocols, patient eligibility criteria, consenting procedures, and sample collection to educate clinicians [38]. This finding highlights that increasing knowledge levels through an educational course on genomics and rGWS is an effective way to address practical barriers affecting implementation.

Many participants in the current study believed that genetics professionals are vital for the successful implementation of rGWS in NICUs. The majority of participants depended heavily on their collaborative relationship with medical genetics when caring for patients undergoing rGWS, regardless of their profession. Further, they believed that geneticists and genetic counselors were the best-suited professionals to implement this technology in NICUs. A strong desire for clinical implementation of rGWS led by genetics has been reported by physicians and geneticists in other NICUs due to inexperience and low confidence levels with rGWS [39]. Further, a recent study examining the attitudes of a multidisciplinary healthcare team about rGWS implementation in PICUs identified that some HCPs strongly believed that genetics professionals should be involved in rGWS implementation [40]. A dependency on medical genetics teams for integrating genomic technology into clinical practices has also been found in other disciplines [36,37]. For example, when examining the integration of genomic sequencing into oncology practices, both Weipert et al. (2018) and Johnson et al. (2017) found that clinicians desired genetic counselors to be involved in the disclosure of genomic sequencing results to patients. Previous studies examining the integration of rGWS into NICUs have highlighted that successful integration requires rGWS champions, who are clinicians who oversee and guide the implementation of this technology in NICUs [36,37]. The results from our study highlight that genetics professionals are the most appropriate champions to guide rGWS implementation in NICUs.

Despite a desire for clinical implementation of rGWS led by genetics teams, participants across all health specialties in this study still believed that certain elements of the rGWS testing process fell within their scope of practice. This is important to note as many participants desired to learn more about rGWS and/or the test results to better support their patients undergoing testing.

Funding applications and delayed turn-around times were identified as operational barriers to rGWS implementation by some research participants. In Canada, healthcare is provincially regulated, and specific eligibility criteria must be met for rGWS approval by the provincial government. This process may delay access to time-sensitive testing, which may impact patient care while waiting for authorization [41]. Participants in the current study expressed frustrations about these delays as they negate the purpose of having a rapid test. These findings provide support to reconsider the approval process/funding applications to reduce operational barriers and promote a more seamless implementation of rGWS into NICUs. Further, they support the need for improved communication between genetics services and the non-genetic HCPs in the NICU about the expected TAT for rGWS results to help manage the expectations of the providers caring for NICU patients undergoing sequencing about when results will be received.

Non-genetic HCPs in NICUs would benefit from the development of resources on rGWS. The development of an electronic “rGWS tracking system” with clear guidelines surrounding sample collection would help address perceived barriers related to sample collection and following test progression. Further, non-genetic HCPs would benefit from being involved in MDT meetings about rGWS.

Additionally, this study extends support for the integration of genetic counselors into NICUs [6]. Genetic counselors have expertise with rGWS and in providing psychosocial support to families navigating genetic testing [42]. In the present study, participants across all specialties expressed concerns and discomfort in their ability to implement rGWS in NICUs and/or support families through the testing process. Previous research has demonstrated that they play a key role in helping NICU parents adapt to genomic information and addressing parental needs for information on the clinical implications of rGWS results [16].

As rGWS becomes more affordable and technological advancements allow for the increased detection of disease-causing variants, arguments are being made to incorporate rGWS into population-based newborn screening programs [43]. Although this could benefit many newborns through early detection, diagnosis, and treatment of genetic diseases [43], the results from this study suggest that non-genetic HCPs may not be sufficiently equipped to support families undergoing rGWS. Before introducing rGWS into other programs such as newborn screening, the barriers identified in this study, including low comprehension levels of genomic sequencing among non-genetic HCPs and discomfort when caring for infants who have undergone rGWS for clinical indications must first be addressed. This research highlights that it is imperative that adequate support and resources for non-genetic HCPs and parents are developed before the widespread implementation of this technology.

### Limitations

The results of this study are not representative of the full diversity of non-genetic HCPs employed in Canadian NICUs as we were unable to interview physical or occupational therapists, medical residents, or fellows. Recommendations for parental support and resources on rGWS are based on the suggestions of the HCPs. Future work should ensure recommendations are evaluated by NICU parents to ensure that they sufficiently meet their support needs.

## 5. Conclusions

This study explored the perspectives of diverse members of multidisciplinary healthcare teams in two Canadian NICUs regarding rGWS. Our findings revealed that non-genetic HCPs support clinical implementation of rGWS led by genetics teams in Canadian NICUs. Further, they showed that non-genetics team HCPs desire additional knowledge, education, and resources to guide the use of rGWS in NICUs. This would help ensure that all members of the NICU multidisciplinary team are supported to help optimize the implementation of rGWS in NICUs.

## Figures and Tables

**Table 1:** Demographic survey data ^1^.

	MD (Neonatologists, Residents, Fellows) (N = 5)	Bedside Nurses (N = 19)	Allied Health Care Providers (RTs, OTs, PTs, Social Workers) (N = 6)
**Years Since Completing Training**			
0–5	3	3	3
6–10	1	7	1
11–15	0	3	0
16–20	1	4	1
20+	0	2	1
**Education Level**			
College Diploma	0	1	0
Bachelor’s Degree	0	16	4
Graduate Degree	1	2	2
Medical Degree	4	0	0
**Self-Reported Genomic Literacy**			
Poor	0	15	4
Average	4	4	1
Very Good/Excellent	1	0	1
**Formal Training in Genetics**			
Yes	2	1	3
No	2	18	3
**Involvement with rGWS patients**			
Never	0	6	0
Sometimes (~2 patients/year)	3	10	3
Frequent (~2 patients/month)	2	3	3

The demographic survey data include data from all participants who completed the survey, including those who completed a semi-structured interview.

**Table 2:** Participants’ quotes highlighting their experiences and perceptions with rGWS, as well as the factors influencing their experiences and perspectives and suggested strategies for the use of rGWS in NICUs.

**Experiences with rGWS**		
**Experience Subcategory**	**Quote in Text ID**	**Representative Quote(s)**
**Exposure to rGWS/genetics**	E1	*“[rGWS] happens so few and far between that you’re actually the one doing it, that you don’t know off the top of your head very well.”* (Nurse, 1 year of experience, ID 10).
**Long turn-around times**	E2	*“I think one of the downsides of the testing […], even though it is rapid, it does take a little bit of time from the start to completion for the results to come in. And so I find that one of the challenges actually is that results do take a little bit longer than what we’re told. And we often have to follow up on things*.” (Neonatologist, 8 years of experience, ID 18).
**Levels of comfort with rGWS**	E3	*“I would be comfortable giving a result if it fit very clearly with the clinical picture and if it’s an expected result, I think I would feel comfortable.”* (Neonatologist, 17 years of experience, ID 5)
	E4	*“I think those situations where you find unexpected findings in your genetic testing, that’s really what makes me uncomfortable.”* (Neonatologist, 8 years of experience, ID 18)”
	E5	*“So I think we don’t have as much information in the NICU to be able to give information [about types of results]… to feel comfortable giving that information to families.”* (Nurse, 6 years of experience, ID 9)
**Communication surrounding rGWS with multidisciplinary team**	E6	*“Genetics is pretty good at bringing us studies on especially those rare conditions. They’ll bring us studies and we’ll have them at our bedside so that if you’re like, “what the heck is this,” and then you can kind of glance it over and read a little bit more.”* (Nurse, 20 years of experience, ID 4).
	E7	*“And to be honest, we don’t get that much information about what’s going on as far as testing for our patients, at least as far as genetic testing goes…And there’s no real drive from people who do understand it that are working on site … to explain it to us, at least not that I’ve seen.”* (Respiratory Therapist, 3 years of experience, ID 22).
**Perceptions of rGWS**		
**Perceptions Subcategory**	**Quote in Text ID**	**Representative Quote(s)**
**More education is needed**	P1	*“I think any education that we can get, any additional education, would be awesome. […] The more we know, the better and easier it is to talk to the families too, and have just a wider knowledge of what could be going on or what we’re looking for.”* (Nurse, 6 years of experience, ID 4).
	P2	*“I feel like I’m always interested to learn more. One thing in particular that would be good to know is the scope and limits of the testing […] so that we can help the family, like support the family through [those] periods of waiting for the testing to come back and understanding what the testing is going to mean, so [the family] can manage their expectations around that.”* (Social Worker, 1 year of experience, ID 32).
**Genetics services are necessary**	P3	*“It would be better suited for genetics professionals to be the ones disclosing those results and counselling the family because they have added knowledge in that area that [I] might not have.”* (Neonatologist, 1 year of experience, ID 28).
	P4	*“But I think oftentimes …. I rely on the genetic counsellors … the team here has a wealth of experience and knowledge about counselling, but also with following up with these patients overtime, and they can provide perspectives that I don’t think we as neonatologists these days can necessarily provide.”* (Neonatologist, 8 years of experience, ID 18)
**rGWS raises ethical concerns**	P5	*“[rGWS] is a little bit of a Pandora’s box. Are we getting more information than we necessarily need? Could we have found it in a simpler way?”* (Nurse, 3 years of experience, ID 25)
	P6	*“You might find a result that might cause more peril, cause more testing, cause … greater anxiety.”* (Neonatologist, 8 years of experience, ID 18).
**rGWS in advantageous**	P7	*“[The quick turn-around-time of rGWS] makes a huge difference especially in the NICU. Because if you have a critically unwell baby and you’re keeping them alive to find out if there’s an illness that you can potentially treat, or there’s one you can’t, that makes a huge difference. Two weeks is a long time to keep a baby in ICU without treatment.”* (Neonatologist, 1 year of experience, ID 28).
	P8	*“Another benefit to having the results in quickly is that the results tend to come back while the baby is still inpatient in the hospital. A baby is only inpatient in the NICU while [there’s] an acute need, and if that testing were to take months to complete, [the baby] may not be inpatient any longer, which means I am no longer supporting them.”* (Social Worker, 1 year of experience, ID 32).
**rGWS impacts parents in the NICU**	P9	*“We’ve had kids that look typical when they are born and then they start breathing weird or they do not follow a typical pattern and then we get their WES back and we find a variant of unknown origin or a secondary factor and then the parents start to question everything.”* (Nurse, 6 years of experience, ID 9).
	P10	*“Alleviating that burden on families of not knowing what’s going on […] is really helpful. In the cases where we get a diagnosis, understanding what other children who have that diagnosis, what their trajectory looks like, is really helpful for families to see what they can expect for the future, and even in the beginning stages of their baby’s life, it’s helpful in terms of determining what [the parents] are going to do.”* (Social Worker, 1 year of experience, ID 32).
**Approval process is unnecessary**	P11	*“But my question is, is this still requiring the funding? It’s the funding that takes a while to be approved. […] All the babies are approved anyway, so what is it about the funding that needs to happen? I just don’t get it.”* (Nurse, 1 year of experience, ID 10).
	P12	*“[Applying for funding] just lengthens the amount of time needed to get the testing done and get the results […] I wish that it was something that you didn’t have to apply for as often.”* (Nurse, 6 years of experience, ID 9).
**Factors Influencing HCPs Experiences and Perspectives with rGWS**		
**Factors Subcategory**	**Quote in Text ID**	**Representative Quote(s)**
**Knowledge level of rGWS**	F1	“*I do know a bit about whole exome sequencing, but I wouldn’t say like I’m an expert on it by any means. Like I’m not 100% sure at what point you’re supposed to order it and what the pitfalls are.”* (Neonatologist, 1 year of experience, ID 28)
	F2	*“It’s kind of embarrassing how little I know on it. And it might be done a lot more than I know, but it’s just kind of going over our heads.”* (Nurse, 6 years of experience, ID 4).
	F3	“*I don’t really know much about the genome sequencing specifically; all I know is it tests for specific genetic conditions.”* (Nurse, 1 year of experience, ID 10).
**Perceived scope of practice and challenges with following test progression**	F4	*“I can disclose results […], but I think once I’ve involved [genetics] they tend to appreciate that part of their job and I don’t want to—you know I want to make sure that I’m not stepping on their toes.”* (Neonatologist, 1 year of experience, ID 28).
	F5	*“The method of testing is really up to a physician discussion, so they are the ones for ordering the tests and then we are just sent to collect blood work.”* (Nurse, 3 years of experience, ID 25).
	F6	*“In my role as a bedside nurse, again, because [dictating management] is so kind of above what we do, it would be genetics with the provider deciding what we would do.”* (Nurse, 6 years of experience, ID 4).
	F7	*“I don’t ever see the reports themselves, and […] I wouldn’t know how to interpret [the results] anyway, it’s more of a conversation about impact.”* (Social Worker, 1 year of experience, ID 32).
**Challenges with following test progression**	F8	*“I remember one time we were trying to figure out if the baby had had testing, and it was such a process…we went through so many hoops to figure out, OK, did the baby have this testing, was it done, was it sent off? Did they approve?”* (Nurse, 1 year of experience, ID 10)
**Strategies for Effective Implementation**		
**Strategies Subcategory**	**Quote in Text ID**	**Representative Quote(s)**
**Resources for HCPs**	S1	*“So I feel like having a system where it’s like, OK, this baby is in the process of funding, this is where we’re at, this is what we’re waiting for, and then a next step, updated sheet thing that OK, the blood has been collected, we’re just waiting for the results to come back. And then a final sheet of paper that says, “Oh, hey, this is the diagnosis, or this is what has come back.” That’s probably, from my area, just to make our jobs easier, and like I said, a little tidbit guideline of what the testing is, how long it takes—just a bit-by-bit piece of information to reiterate to families.”* (Nurse, 1 year of experience, ID 10)
**Resources for families**	S2	*“It’s a stressful time in the NICU, and getting all that information could have negative psychological effects. But having appropriate supports like social workers or genetic counsellors there to help with that would help mitigate those risks.”* (Respiratory Therapist, 3 years of experience, ID 22).
**Education for HCPs**	S3	*“Having a series of lectures updating us on the latest [rGWS] technologies, pros, cons, limitations, benefits … I think that would be very interesting and I’d love to listen to those.”* (Neonatologist, 8 years of experience, ID 18).

**Table 3:** Recommended components to inform a guideline to help integrate rGWS into Canadian NICUs.

Suggested Guidance for rGWS Implementation in NICUs
1. A clear description of the rGWS eligibility criteria and when testing is appropriate
2. An outline of which HCPs are responsible for which components of the rGWS (collecting the blood sample, consenting, disclosing results, patient follow-up, etc.)
3. An outline of the logistics surrounding sample collection (type of tube, how much blood to collect, where to send the sample, etc.)
4. Informed pre-test counseling for families, including a discussion of the scope and limitations of the testing to manage parental expectations
5. A description of what elements of rGWS should be discussed with families when obtaining informed consent
6. A description of the potential types of results and what results are reported to families
7. The requirement for families undergoing rGWS to be equipped with support and resources
8. The opportunity for families to have a follow-up appointment with the medical genetics team shortly after receiving their rGWS results and being discharged

## Data Availability

The data presented in this study are available on request from the corresponding author. The data are not publicly available due to privacy or ethical considerations.

## Supplementary Materials

The following supporting information can be downloaded at: https://www.mdpi.com/article/10.3390/children11080910/s1, S1: Demographic Survey; S2: Interview Guide.
