# Supplementary material for: Non-Genetic Healthcare Providers’ Experiences and Perspectives with Rapid Genome-Wide Sequencing in Canadian Neonatal Intensive Care Units
Source: Children (Basel). 2024 Jul 28;11(8):910. doi: 10.3390/children11080910 (PMC11352610; doi:10.3390/children11080910)
Supplement: Supplementary file 1 [file children-11-00910-s001.zip › children-3114363-supplementary.pdf]

## Supplementary Materials

### Demographic Survey S1

**Study Title:** Exploring healthcare providers' experiences and perspectives of rapid genome-wide sequencing for critically ill infants in British Columbia NICU settings.

Thank you for taking this initial brief survey about your experiences and perceptions of rapid genome wide sequencing (rGWS) for critically ill infants in the NICU. The purpose of this survey is to gather some brief demographic information and determine your experience with rGWS.

Completing and submitting your response to this survey will be considered as consent to use your data in our analysis. If you are willing to participate in the second part of the study (i.e., 50 minute online interview or telephone interview), please be sure to include your email address at the end of the survey. We are asking to collect your email address because you have agreed to be contacted to be interviewed. Although you may not be aware of this fact, emails sent to some webmail services (e.g. Gmail, Hotmail, etc.), may be stored/routed outside of Canada (for example, in the United States). Due to the fact that future emails will contain personal information about you, including your name, the Freedom of Information and Protection of Privacy Act (FIPPA) requires that we obtain your consent before we continue. We will only send your personal information to the email address you have provided to us. All of the information which you provide to us will be kept completely confidential. Providing your email address means that you voluntarily agree and give your consent for the study team to email your personal information to you.

Please feel free to omit any questions that you feel may make you identifiable.

| | |
|---|---|
| Healthcare provider specialty | Neonatologist Nurse Respiratory Therapist<br>Social Worker Medical Fellow/Resident<br>Occupational Therapist Physical Therapist Dietician<br>Pharmacist Other |
| Specialty: Specify “Other” | |
| Education Level | Bachelor Degree Graduate Degree Medical Degree<br>Other |
| Education Level: Specify “Other” | |
| For non-trainees: Years since the completion of training | |
| How would you rate your genomic literacy? | Excellent Very Good Average Poor |
| Have you ever received any formal training in genetics? | Yes No |
| If yes, please specify | |
| How often are you involved in the care of patients who have had rapid genome wide sequencing (rGWS)? | Never Sometimes (2/year) Frequently (~2/month) |
| If you are willing to be contacted for a follow up interview, please provide your contact information: | |
| Email address | |
| Alternative contact | |

## Interview Guide S2

**Study Title:** Exploring healthcare providers' experiences and perspectives of rapid genome-wide sequencing for critically ill infants in British Columbia NICU settings

**General Experiences:**

1. Tell us about your experiences with rapid genome wide sequencing (rGWS).
   - Prompts: Have you provided care of infants who have been genetically tested using rGWS? Would you say they were positive or negative experiences? Please, explain.
2. How comfortable do you feel deciding when testing is appropriate (for neonatologist/medical fellow/resident)?
   - Prompt: What factors do you consider when deciding whether it is appropriate?

**Clinical and Ethical Aspects:**

1. *The rGWS testing process consists of many elements, from discerning which infants should undergo rGWS, to obtaining consent, interpreting results, and sharing the results with the family.* What aspects of the rGWS testing process do you feel comfortable with?
   - Prompts: How confident do you feel in your level of understanding of rGWS? How comfortable do you feel obtaining consent from parents/families for their children to undergo rGWS? What are the critical elements of the test that should be discussed with the families to obtain their consent?
   - Prompt (for neonatologists/medical fellows/residents)?: How comfortable did you feel interpreting the results of the tests? Prompt (for other HCPs not ordering the test): When the results from the rGWS were shared with you, how comfortable did you feel with understanding the implications of the test results to be able to care for the infant and support their parents?
   - Prompts: How comfortable did you feel modifying the treatment plan because of the results of the tests? How comfortable did you feel discussing the test results with the families? With your colleagues? What questions do parents/families frequently/commonly ask about their child's results? How comfortable do you feel answering the questions of the patients/families? What difficulties have you experienced in your practice or anticipate you may experience regarding incidental findings and secondary findings? How confident do you feel addressing these issues?
2. How can the process of obtaining consent for the test be improved?
3. How does using rGWS in the NICU impact the care you provide to the newborn?
   - Prompts: Can you give us specific examples? Has this been different compared to the care you provided using traditional genetic testing?
4. What concerns, if any, do you have about potential insurance implications rGWS might create?
   - Prompt: Are you concerned about potential threats to confidentiality?
5. What concerns have you had regarding your patient's (and their family's) psychological well-being?

**Practical Barriers and Facilitators:**

1. What are the potential benefits and drawbacks of introducing rGWS as a first-tier test in the NICU?
2. What resources do you need at the point of care to successfully implement rGWS?
   - Prompt: Would you find it useful for HCPs to receive additional training in genomics?
3. *Traditionally, genetic testing results could take several weeks or even months to become available. Now that rapid genome wide sequencing has been implemented, results can be obtained in 7 days.* How has this reduction in turnaround time impacted the way you engage with this type of information?
4. What critical elements do you think should be included in a practice guideline about rGWS?
5. How does the process of having to apply for funding from the government and wait for approval impact your ability to offer rGWS?

**Closing Remarks:**

1. Are there any additional comments that you would like to share with us?
